# Auxiliary-assisted chemical ubiquitylation of NEMO and linear extension by HOIP

**DOI:** 10.1038/s42004-019-0211-7

**Published:** 2019-09-19

**Authors:** Fabienne Burlina, Abu-Baker M. Abdel-Aal, Richard Raz, Irene Pinzuti, George Papageorgiou, Jiejin Li, Robin Antrobus, Stephen R. Martin, Simone Kunzelmann, Benjamin Stieglitz, John Offer

**Affiliations:** 1 Sorbonne Université, École normale supérieure, PSL University, CNRS, Laboratoire des biomolécules, LBM, 75005 Paris, France; 2 The Francis Crick Institute, 1 Midland Road, London NW1 1AT, UK; 3 Department of Biochemistry, School of Biological and Chemical Sciences, Queen Mary University of London, Mile End Road, London E1 4NS, UK; 4 Cambridge Institute for Medical Research, Cambridge Biomedical Campus, Wellcome Trust/MRC Building, Hills Road, Cambridge CB2 OXY, UK

## Abstract

The ubiquitylation of NF-κB essential modulator (NEMO) is part of the intracellular immune signalling pathway. Monoubiquitylated NEMO is required for exploring the mechanism of NEMO linear ubiquitylation by LUBAC (linear ubiquitin chain assembly complex), but is not accessible by biological techniques. Here we perform the chemical ubiquitylation of NEMO using a ligation auxiliary, which only requires a two-step synthesis, and is easily installed onto the lysine side-chain. Chemical ligation occurs directly on the lysine ε amine and remains efficient below pH 7. We show that ubiquitylated NEMO has similar affinity to linear diubiquitin chains as unmodified NEMO. The proximal ubiquitin of chemically synthesised NEMO_CoZi_-Ub is accepted as a substrate for linear extension by the (RING-Between-RING) RBR domain of HOIL-1-interacting protein (HOIP) alone. Our results indicate that NEMO linear ubiquitylation consists of two-steps, an initial priming event and a separate extension step requiring different LUBAC components.

Intracellular immune signalling is largely mediated by post-translational modification of proteins with ubiquitin (Ub) chains^1^. In particular, linear (or M1-linked) Ub chains promote activation of the NF-κB (nuclear factor ‘kappa-light-chain-enhancer’ of activated B-cells) pathway, a key event in mounting an inflammatory response to bacterial and viral infections^2^. NF-κB essential modulator (NEMO) is the regulatory subunit of IKK (inhibitor of nuclear factor kappa-B kinase), a kinase complex, which tightly regulates NF-κB. Linear ubiquitylation of NEMO plays an essential role by promoting activation of IKK. NEMO is a dimeric 48 KDa protein, containing many linear ubiquitylation sites, mostly in the CoZi (coiled-coil and leucine zipper) domain^3^. The CoZi domain is also the site that binds linear Ub chains with high specificity, required for the regulatory function of NEMO.

The linear ubiquitin chain assembly complex (LUBAC), is the only known ligase complex to synthesise linear ubiquitin. LUBAC is a hetero complex consisting of the three proteins HOIP (HOIL-1-interacting protein), HOIL-1 (Heme-oxidized IRP2 ubiquitin ligase 1) and SHARPIN (Shank-associated RH domain-interacting protein) (Fig. 1). HOIP and HOIL-1 display linear Ub chain synthesis activity via their RING-Between-RING (RBR) domains^4^. HOIP is the major catalytic core of LUBAC and essential for linear ubiquitin chain synthesis^5–7^. The isolated C-terminal RBR domain of HOIP can synthesise free, unanchored Ub chains, whereas HOIL-1 displays only low basal chain assembly activity^6^. However, HOIL-1 is required for releasing autoinhibition of HOIP and might be required for ab initio ubiquitylation of NEMO^8^. Similar to HOIL-1, SHARPIN is also able to release autoinhibition, but does not display ubiquitylation activity. It has been proposed that HOIL-1 directs HOIP catalytic activity so that the initial donor Ub is transferred onto NEMO^8^. The molecular determinants for subsequent rounds of linear Ub chain elongation have not been established. So far it remains elusive if this reaction requires the entire LUBAC complex or if the HOIP catalytic core is sufficient for linear ubiquitylation of Ub primed NEMO. In order to investigate LUBAC mediated ubiquitylation of NEMO we wished to reconstitute the cascade for linear ubiquitylation including monoubiquitylated NEMO_CoZi_ (NEMO_CoZi_-Ub) as a substrate. While the reconstitution of the ubiquitylation cascade for free unanchored Ub chains is established by using purified proteins from bacterial expression systems, the investigation of linear chain synthesis in context of a substrate protein such as NEMO was not previously conceivable because monoubiquitylated NEMO was not obtainable from current biological expression systems.

Chemical protein synthesis enables a virtually limitless choice of functionalities at defined positions and has evolved into a powerful technique for exploring the biological role of post-translational modifications (PTMs), notably ubiquitylation^9–11^. There are several approaches used to form the native isopeptide attachment. The most widely used methods are extensions of native chemical ligation (NCL); including the use of δ-thiolysine (in both synthetic^12^ and recombinant proteins^13^) and γ-thiolysine^14^, or the use of a ligation auxiliary, removed after ligation by photolysis^15,16^, acidolysis^17^, or reduction^18^. Auxiliaries are notorious for being practically limited to only the most sterically unhindered ligation junctions. From steric considerations, formation of the isopeptide bond would appear to be ideal for the auxiliary approach but in practice ligation has usually been performed at one remove from the isopeptide bond between the two glycines of ubiquitin. This is probably because of the high pka of the lysine side-chain and therefore ubiquitylation is largely prevented at neutral pH as the lysine side-chain amine is protected by protonation. The 1-(2,4-dimethoxyphenyl)-2-mercaptoethyl auxiliary^17^ has been the most widely used auxiliary for ubiquitylation and many impressive targets have been successfully prepared with it^19–22^. However, as also for the mercaptolysine approach, it requires multi-step synthesis to prepare the protected building blocks and several equivalents are required for peptide synthesis. Some alternative approaches have overcome these difficulties by avoiding the use of an auxiliary or mercaptolysine^23,24^.

For our synthesis of NEMO_CoZi_-Ub, we wanted an easily synthesised auxiliary that could be installed directly onto the lysine ε-amine post-synthesis (Fig. 2). In contrast the current auxiliary approaches for ubiquitylation couple a fully-protected auxiliary derivatized amino acid building block. The 2,3,4-trimethoxymercaptobenzyl (Tmb) auxiliary has been widely applied to the synthesis of proteins and conjugates^25–29^. Unlike other comparable auxiliaries such as 1-(2,4-dimethoxyphenyl)-2-mercaptoethyl or thiol-derivatized lysine, it has no chiral centre (and therefore no accompanying HPLC peak-splitting or broadening) and can be used in the presence of unprotected cysteines^28^. Tmb, like other auxiliaries, is generally added as a fully-protected building block^19^ (auxiliary attached to a protected amino acid). This is because aldehydes generally undergo inefficient reductive amination on solid phase making this approach only really suitable for short peptides^26^. Salicylaldehydes, however, are an exception, forming unusually stable imines^30^ and a single equivalent of salicylaldehyde is generally adequate for quantitative reaction on solid-phase^31^. Simple demethylation of the ortho position of Tmb aldehyde converts it to a salicylaldehyde thereby assisting its easy installation onto a primary amine. Additionally, we anticipated that the ortho positioning of the hydroxyl could potentially assist S to N acyl transfer by intramolecular assistance of the proton transfer step in the ligation mechanism^32^. An ortho hydroxyl assists the reverse, N to S acyl migration^33^.

Here we show that the combination of optimised solid-phase synthesis of long peptides and an auxiliary-assisted ubiquitylation to form the isopeptide attachment enables the chemical preparation of monoubiquitylated NEMO_CoZi_. This allows us to explore the linear ubiquitylation pathway by directly testing if HOIP can extend a linear Ub chain on NEMO_CoZi_-Ub in the absence of HOIL-1 and measure the ubiquitin chain binding affinity of ubiquitylated NEMO.

## Results

### Auxiliary synthesis and installation

**Fig. 2:**
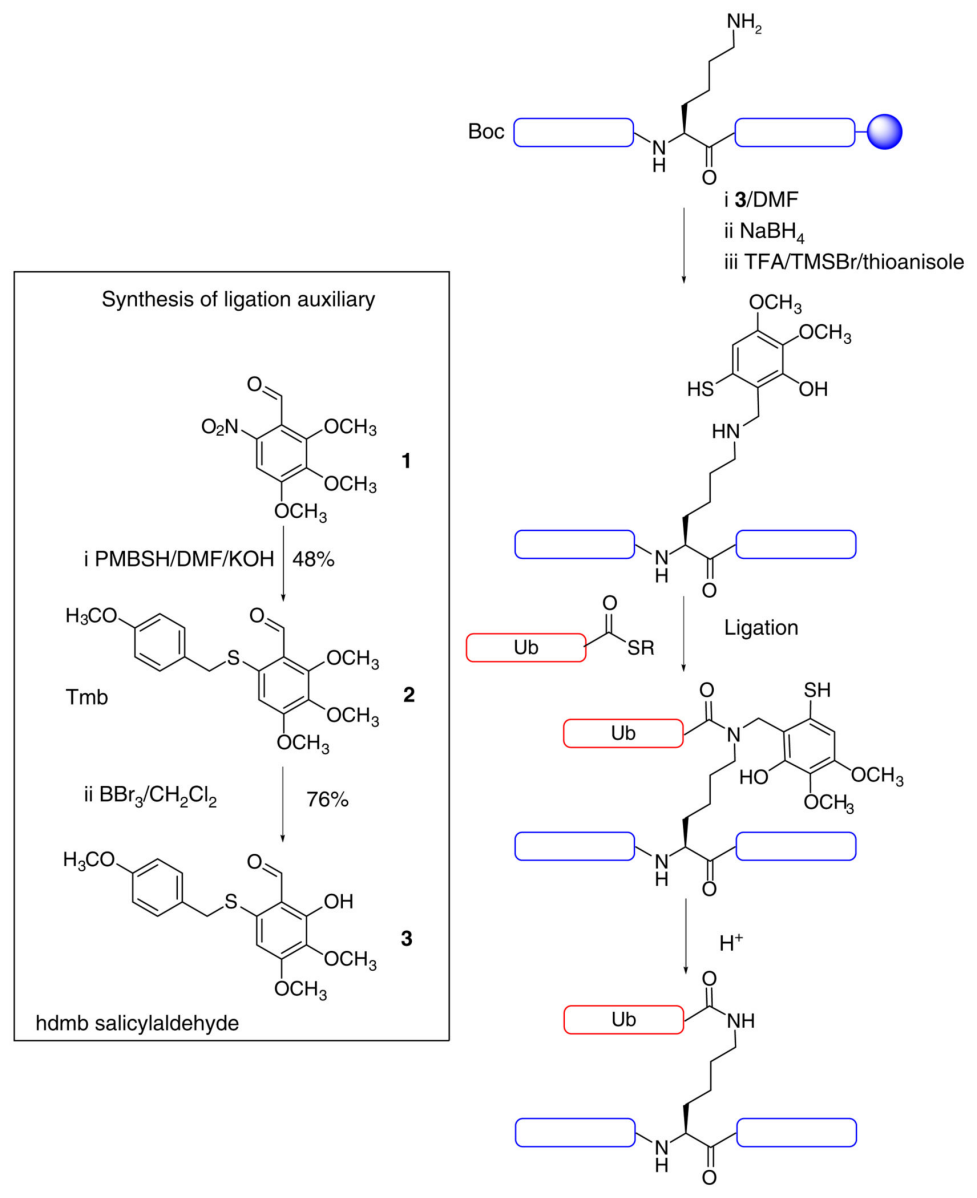
General strategy of hdmb auxiliary-assisted ubiquitylation. Synthesis of the hdmb salicylaldehyde ligation auxiliary **3**. Installation of the hdmb salicylaldehyde auxiliary onto the lysine side-chain of the fully-protected peptide-resin, ubiquitylation and hdmb auxiliary removal

One obstacle to the use of ligation auxiliaries has been the multi-step synthesis required^23^. Therefore we first optimised and shortened the synthesis of the Tmb aldehyde **2** to a single easy step. It was prepared in 48% yield by substituting the nitro group of 2,3,4-trimethoxy-6-nitro-benzaldehyde **1** with *p-*methoxybenzylmercaptan in the presence of KOH (Fig. 2)^19,28^. Tmb aldehyde **2** was converted to 2-hydroxy-3,4-dimethoxy-6-(4-methoxybenzylthio)benzaldehyde **3** in 76% yield by chemoselective demethylation of the 2-methoxy group. The salicylaldehyde auxiliary **3** could therefore be prepared in only two steps from commercially available nitro-benzylaldehyde **1**. Importantly, there was no need to subsequently synthesize a bespoke fully-protected building block.

**Fig. 3:**
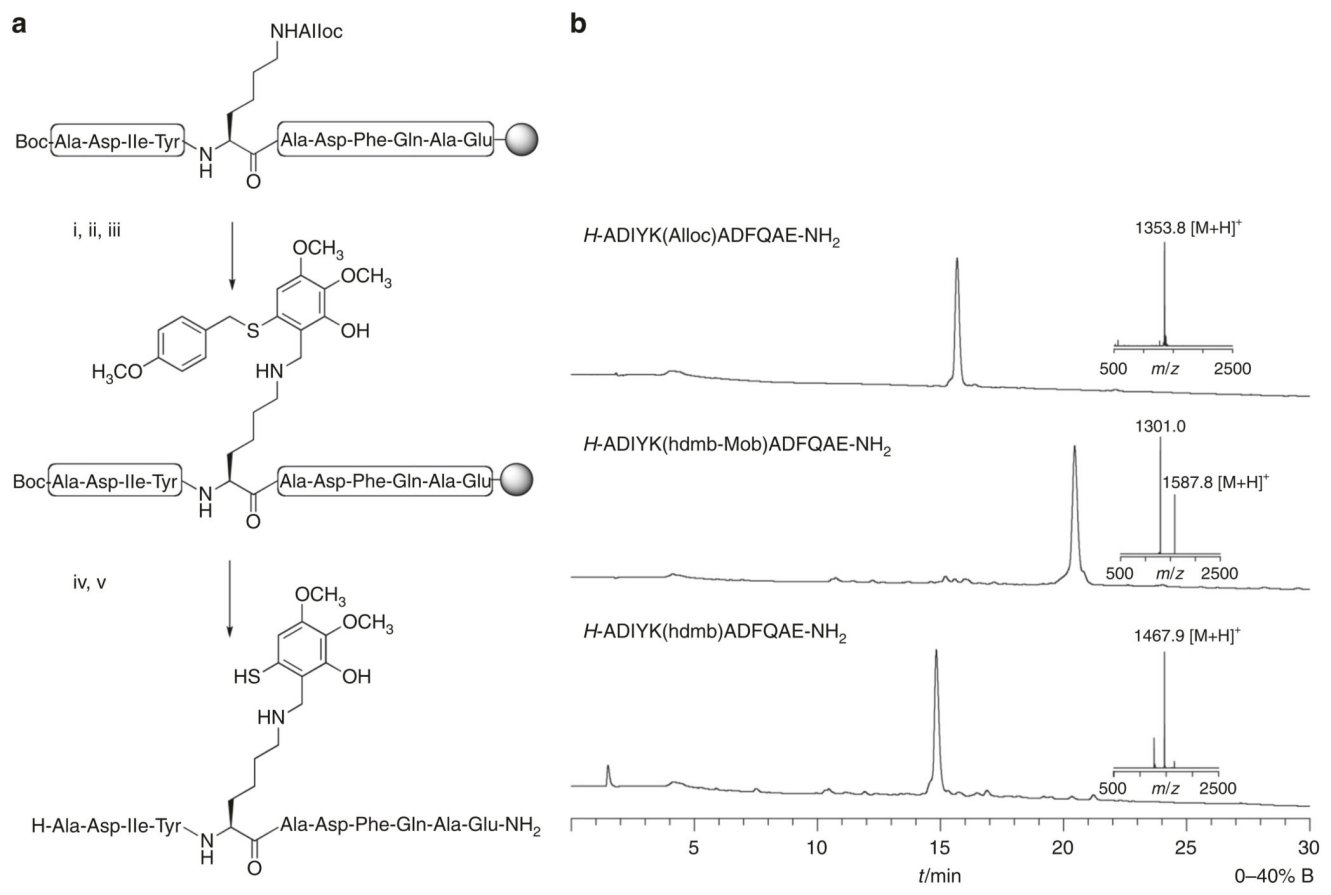
Installation of the ligation auxiliary hdmb onto NEMO_298–308_. **a** Scheme for direct addition of the auxiliary to the lysine side-chain, conditions: (i) Pd (PPh_3_)_4_, PhSiH_3_, DCM (ii) 3, DMF (iii) NaBH_4_, DMF (iv) TFA/TES/EDT/H_2_O (v) TFA/TMSBr/thioanisole/EDT. **b** Analytical HPLC of crude peptides and MALDI-TOF MS of pure peptides at stages of auxiliary addition. *H*-ADIYK(Alloc)ADFQAE-*NH_2_*: *m/z* = 1353.8 [M + H]^+^, Calc. = 1353.6. *H*-ADIYK(hdmb-Mob)ADFQAE-*NH_2_*: m/z = 1587.8 Calc. = 1587.7 (a metastable ion is also observed *m/z* = 1301.0). *H*-ADIYK(hdmb)ADFQAE-*NH_2_*: *m/z* = 1467.9 [M + H]^+^, Calc. = 1467.6

The addition of hdmb auxiliary was first attempted on a small model peptide (for easy monitoring by HPLC) corresponding to a fragment of the CoZi domain of mouse NEMO (NEMO_298–308_, ADIY**K**ADFQAE) and containing the Lys^302^ residue targeted for ubiquitylation (Fig. 3). The Lys side-chain was deprotected and a single equivalent of hdmb salicylaldehyde **3** added to the swollen peptide-resin and reduced. NEMO_298–308_hdmb was cleaved from the resin and the Mob group removed. Mob is a versatile protecting group removable under several conditions, in this case TMSBr was used^26,34^. Analytical HPLC of crude peptide samples at each step indicated that hdmb addition was complete and therefore potentially suitable for long peptides (Fig. 3).

### Auxiliary-mediated ligations

We explored the ligation properties of hdmb derivatised peptides with the simplest model system, NEMO_298–308_hdmb and the test peptide thioester LAPAG-MPAL. Ligation in denaturing phosphate buffer pH 7 was complete after 6.5 h (Supplementary Fig. 1). In contrast, the ligation of NEMO_298–308_Tmb to the test peptide under the same conditions of pH and concentration gave no product. After lyophilisation of the ligation mixture, the auxiliary was removed by acidolysis with no reverse N to S rearrangement detected, a concern for any extended ligation method^27^. The product was isolated in a combined yield for the two steps (ligation and auxiliary removal) of 55%. This result encouraged us to repeat the reaction with ubiquitin.

**Fig. 4:**
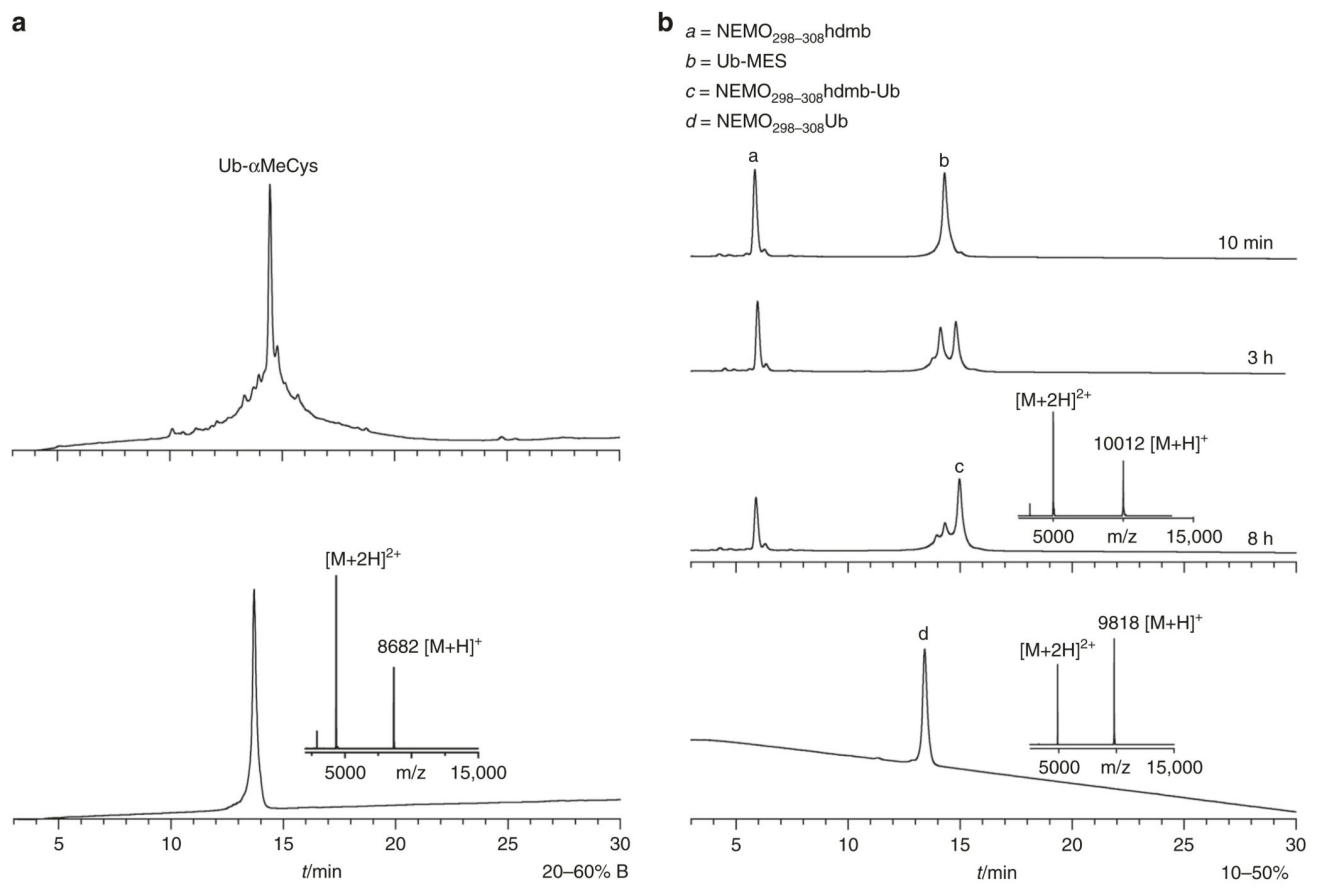
Synthesis of ubiquitin-αMeCys and model ligation. **a** Analytical HPLC (RP-C18) of crude and purified Ub-αMeCys. MALDI-TOF MS, *m/z* = 8682 [M + H]^+^, calc. Av. = 8682. **b** Model ligation of NEMO_298–308_hdmb with Ub-MES thioester, time-course monitoring of ligation reaction by analytical HPLC, RP-diphenyl. Bottom, analytical HPLC of NEMO_298–308_-Ub (after hdmb removal) (d) and MALDI-TOF MS, *m/z* = 9818 [M + H]^+^, calc. Av. = 9817

Full-length Ubiquitin_1–76_ (Ub) was synthesized stepwise on αMeCys loaded Wang resin^35^ using pseudoproline and Hmb backbone substitution positioned throughout the sequence after Ovaa and co-workers^36^. The synthesis gave a crude Ub-αMeCys material of excellent quality and was purified in 28% isolated yield (Fig. 4a). α-MeCys undergoes N to S acyl transfer in presence of thiol additives to generate a thioester^37^. Thioester exchange with MESNa thiol to give Ub-MES thioester was complete in under 6 h (90% isolated yield). The exchange reaction was repeated with MPAA thiol to give the more activated Ub-MPAA thioester (Supplementary Fig. 3). Next we carried out ligation of the model sequence NEMO_298–308_Hdmb with Ub-MES thioester in denaturing phosphate buffer at a final pH of 6.5. The ligation was practically complete around 8 h (Fig. 4b). Ligation was repeated under the same conditions with Ub-MPAA and, as expected, it was faster, complete in 2 h. The auxiliary was cleanly removed by acidolysis.

**Fig. 5:**
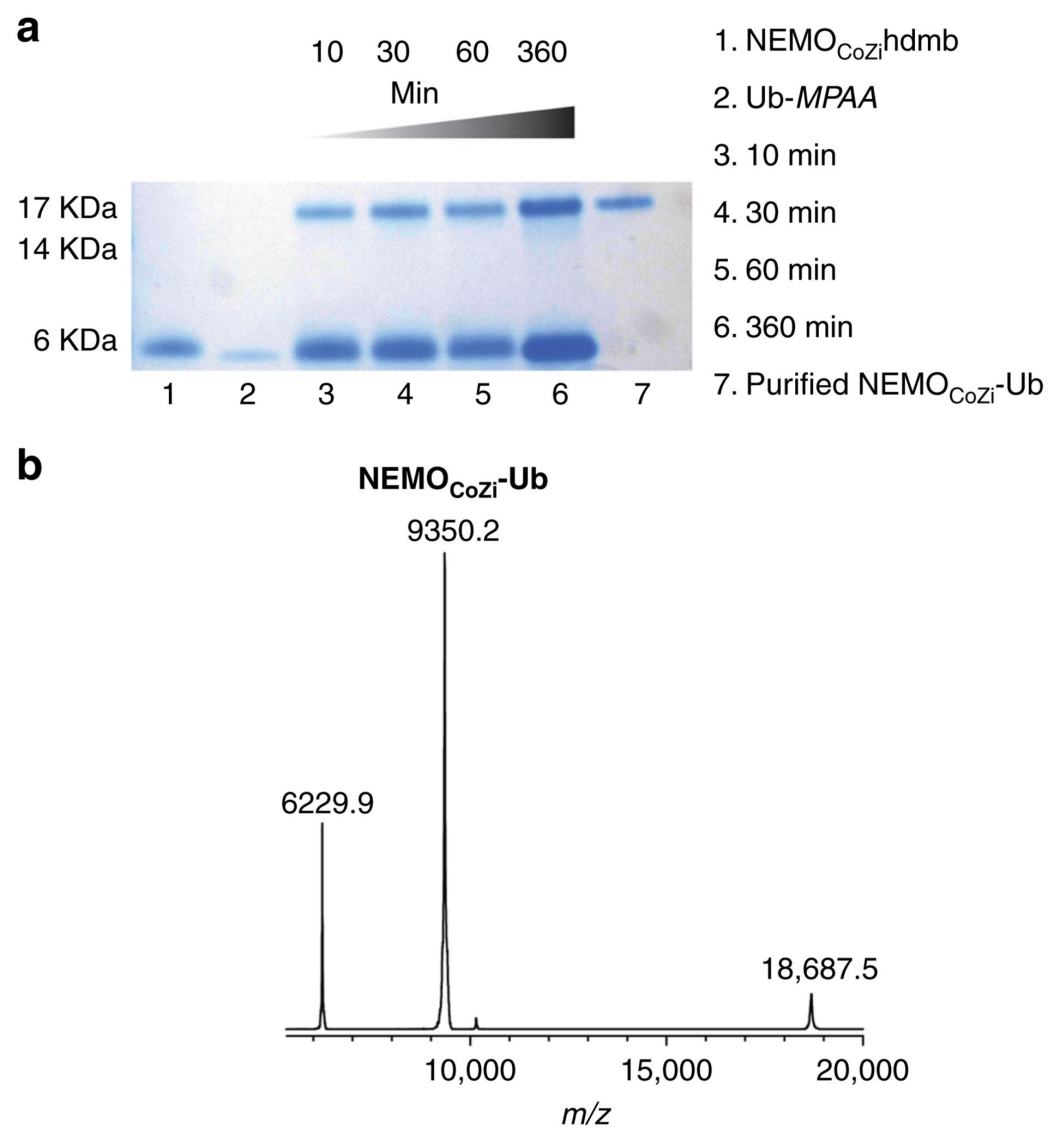
Chemical ubiquitylation of NEMO_CoZi_ with hdmb auxiliary-assisted ligation. **a** Time-course for auxiliary ubiquitylation of NEMO_CoZi_ monitored by gel electrophoresis (lane 1–6). Lane 7 is the ligation product after hdmb removal and HPLC purification. **b** MALDI-TOF MS of NEMO_CoZi_-Ub *m/z* = 18688 [M + H]^+^, calc. Av. *m/z* = 18690

With the success of the model ligations, which showed that the hdmb auxiliary could promote efficient ligation on a Lys side-chain even below pH 7, we addressed our goal, the ubiquitylation of mouse NEMO CoZi domain (NEMO_CoZi_)(PDB ID:3F89). Lys^302^ corresponding to human NEMO Lys^309^ has been identified as a position that is linearly ubiquitylated^38^ and also the target of bacterial ligase activity to switch off NEMO^39^. The structure of (NEMO_CoZi_)(PDB ID:3F89) as a dimer has been reported and was chosen as an ideal target for synthesis because of its size^40^. The 83-mer NEMO_CoZi_ was synthesized in a single piece with Lys^302^ side-chain protected with Alloc and an N-terminal biotin. After Alloc removal (Supplementary Fig. 5), the hdmb salicylaldehyde **3** was added and reductive amination, cleavage and Mob removal performed satisfactorily as established previously for the model peptide (Supplementary Figs. 6 and 7). Chemical ubiquitylation of NEMO_CoZi_ was first performed with Ub-MES. Initially the ligation proceeded well; however, over the course of the ligation (after overnight reaction), the product aggregated. As observed previously ubiquitylation can dramatically change protein solubility^41,42^ and ubiquitylated NEMO_CoZi_ appeared to be much less soluble in the ligation conditions than either of its precursors. The ligation was therefore repeated with Ub-MPAA to speed up the reaction and thereby avoid aggregation. Ligation at final pH 6.5 using 1 mM NEMO_CoZi_hdmb and 2 equivalents of Ub-MPAA was complete in under 6 h without aggregation. The ligation was monitored by gel electrophoresis (Fig. 5a) and hdmb removed by direct acidolysis of the crude freeze-dried ligation mixture. HPLC purification yielded NEMO_CoZi_-Ub in 38% yield for combined steps of ligation and auxiliary removal, and gratifyingly MALDI-TOF MS gave the expected mass (Fig. 5b). The specificity of the ubiquitylation was proven by trypsin digestion and subsequent LC-MSMS analysis. The distinctive GG tag confirmed ubiquitylation at position Lys 302 (Supplementary Fig. 8).

### Folding and di-ubiquitin chain binding of NEMO_CoZi_ derivatives

**Fig. 6:**
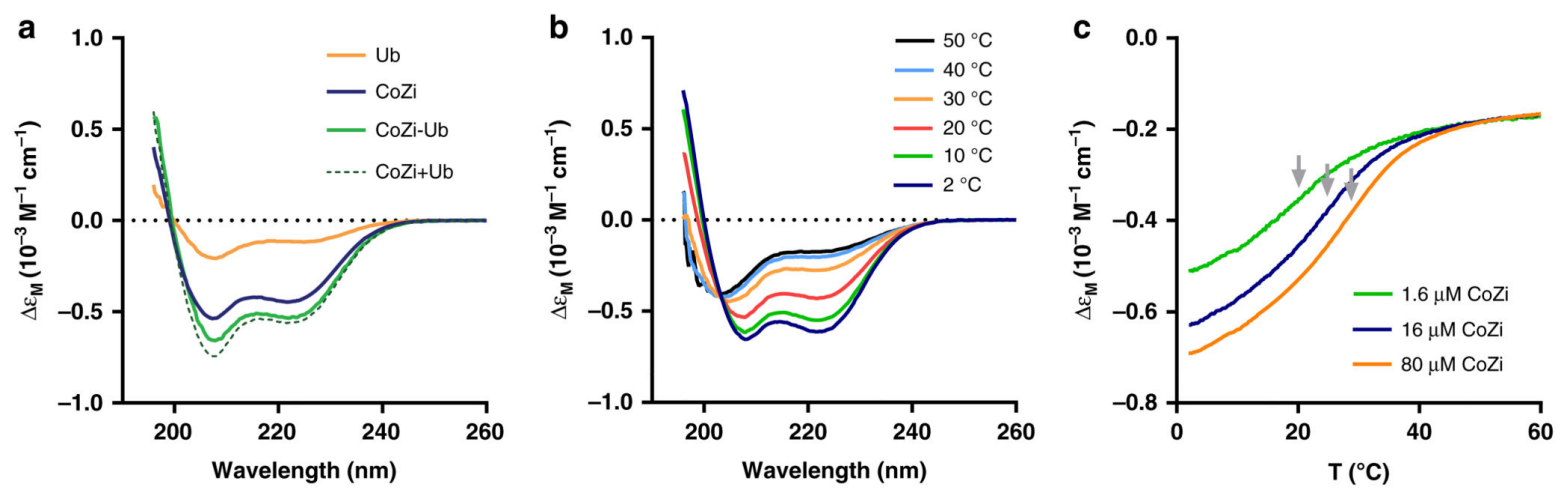
Structural characterisation of NEMO_CoZi_ and NEMO_CoZi_-Ub by circular dichroism spectroscopy. **a** Far-UV CD spectra of NEMO_CoZi_, NEMO_CoZi_-Ub and ubiquitin measured at 20 °C. The CD spectra of NEMO_CoZi_-Ub is very similar to the the sum of the spectra of the individual components, NEMO_CoZi_ and ubiquitin. **b** Far-UV CD spectra of NEMO_CoZi_ (16 μM) at different temperatures. **c** Thermal melting curves of NEMO_CoZi_ measured at 222 nm. The melting temperature (grey arrows) increases at higher concentrations of NEMO_CoZi_, from 20.2 °C, to 24.9 °C, and to 28.5 °C

The folding of synthetic NEMO_CoZi_ derivatives was studied by circular dichroism. NEMO_CoZi_ displayed far-UV CD spectra characteristic of helical secondary structure (Fig. 6a), which converts to a random coil at elevated temperature (Fig. 6b). To demonstrate that the α-helical structure of NEMO_CoZi_ coils together to form a dimer we determined the melting point of the protein at different concentration (Fig. 6c). The melting temperature is clearly shifting with increasing concentrations of NEMO_CoZi_, indicating that synthetic biotinylated NEMO_CoZi_ forms a coiled-coil dimer consistent with the crystal structure derived from bacterially expressed NEMO_CoZi_ (PDB: ID 3F89)^3,40^.

We also determined CD spectra of synthetic ubiquitylated NEMO_CoZi_, which displays additional secondary structure to NEMO_CoZi_ alone (Fig. 6a). The CD spectra of NEMOCoZi-Ub closely resembles the sum of the two separate spectra of NEMO_CoZi_ and ubiquitin, indicating that both parts, the NEMO coiled-coil as well as the ubiquitin are properly folded. Altogether, the circular dichroism data strongly suggest that our synthetic NEMO_CoZi_ derivatives adopt the native fold of their endogenous counterparts.

**Fig. 7:**
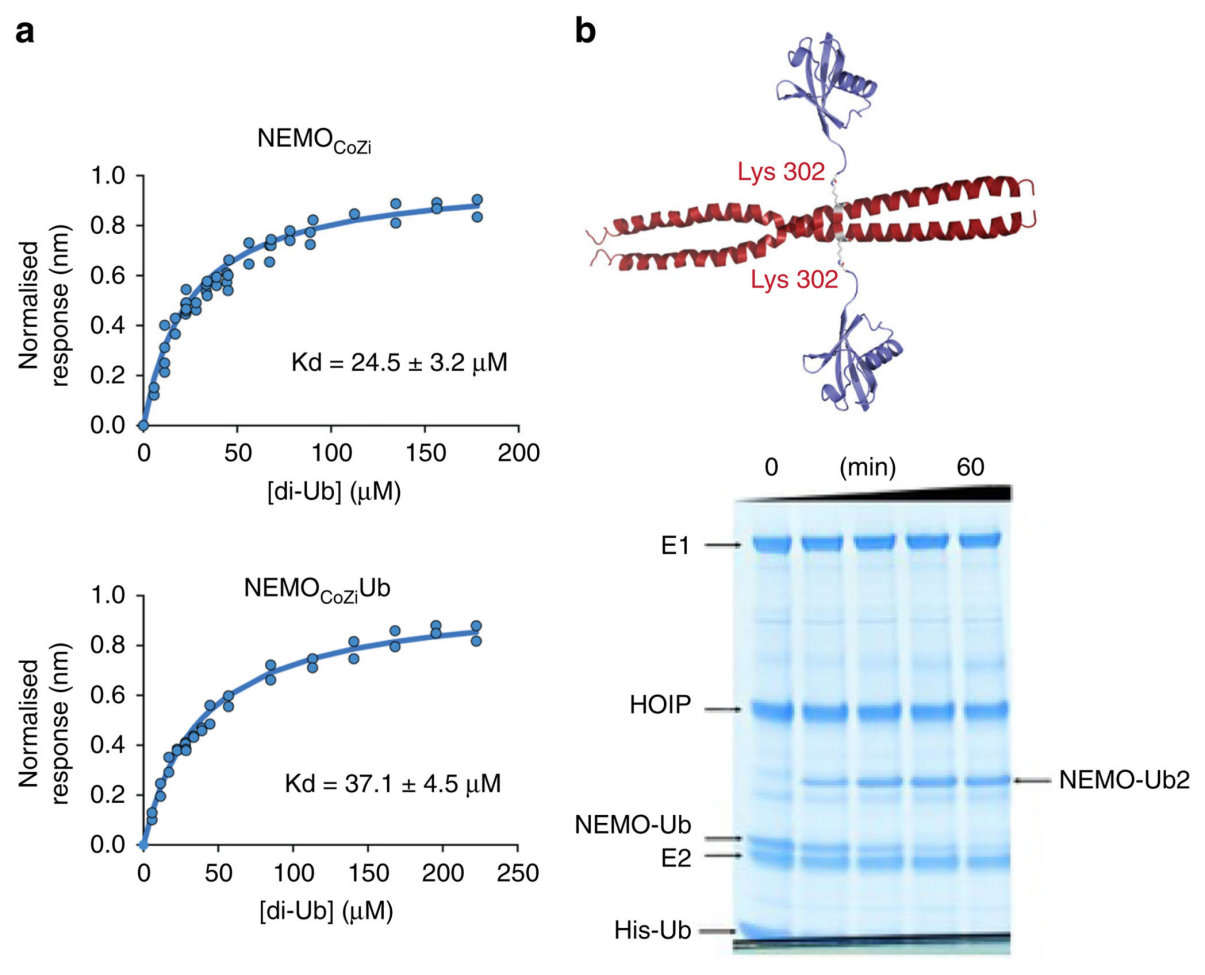
Biophysical characterisation and substrate properties of NEMO_CoZi_-Ub **a** Biolayer interferometry measurement of binding of linear di-Ub to NEMO_CoZi_ and NEMO_CoZi_-Ub. Data points from four independent experiments are shown. Errors on the dissociation constants represent the standard error from non-linear least square fitting to the combined datasets. **b** model of the NEMO_CoZi_-Ub coiled-coil, time-course of linear ubiquitylation of NEMO_CoZi_-Ub by the RBR domain of HOIP (HOIP) and E1 and E2 ligases with N-terminal His-tagged ubiquitin (His-Ub) showing the emergence of di-ubiquitylated NEMO_CoZi_ (NEMO-Ub2)

NEMO CoZi domain is not only a substrate for ubiquitylation, but also binds linear Ub chains with high affinity^3,40^. It is believed that linear Ub binding of NEMO is required for recruitment of IKK into the TNF receptor-signalling complex (TNFRSC), which results in ubiquitylation of NEMO by LUBAC and triggers subsequent kinase activation of IKK. However, little is known about the impact of NEMO linear ubiquitylation on non-covalent complex formation. With the biotinylated synthetic material we could directly measure and compare the affinities of NEMO_CoZi_ and NEMO_CoZi_-Ub for linear di-Ubiquitin (di-Ub). Both NEMO derivatives were immobilized via their N-terminal biotin and dissociation equilibrium constants were measured by biolayer interferometry. It was found that NEMO_CoZi_ interacts with linear di-Ub with a K_d_ of 25 μM (Fig. 7a). This value is about 10-times higher when compared with the K_d_ value obtained by isothermal titration calorimetry using the NEMO CoZi domain from bacterial expression systems (K_d_ = 2.4 μM)^3^. The difference possibly reflects the use of distinctive techniques (which involve immobilization or not) and the use of CoZi domain constructs of different length. Interestingly, monoubiquitylated NEMO_CoZi_ has only slightly lower affinity for linear di-Ub (K_d_ = 37 μM) compared to unmodified NEMO_CoZi_.

### Ubiquitin elongation of NEMO_CoZi_-Ub by the RBR domain of HOIP

Next, we set out to define the minimal catalytic complex of LUBAC required for linear ubiquitin chain elongation of NEMO primed with Ub. In order to probe if NEMO_CoZi_-Ub can be utilised as a substrate we used purified proteins (RBR domain of HOIP, E1 and E2 ligases) from bacterial expression systems to reconstitute the ubiquitylation cascade. We used His-tagged Ub as a substrate for the chain synthesis reaction with NEMO_CoZi_-Ub. His-tagged Ub, is sterically blocked at the N-terminus and can only be utilised by LUBAC as donor Ub for chain elongation, but not for the synthesis of free, unanchored Ub chains. The enzymatic reaction was monitored by gel electrophoresis (Fig. 7b). The formation of di-ubiquitylated NEMO_CoZi_ was observed showing that ubiquitin elongation is performed by HOIP alone and does not require HOIL-1 and SHARPIN.

## Discussion

Our original goal was to demonstrate the practicality of our new ligation auxiliary hdmb on a challenging biological question. This was accomplished by the successful synthesis of NEMO_CoZi_-Ub. We established an expedited synthesis of hdmb salicylaldehyde (two steps) and showed its efficient post-synthesis installation onto a long peptide, the size of a typical protein domain. The ligation of two large fragments, full-length synthetic ubiquitin and NEMO_CoZi_, at low mM concentration, was complete on a practical time-scale (6 h), which was critical in this case because of the aggregation-prone product. Interestingly, hdmb ligation occurred comfortably below pH 7, in contrast to other auxiliaries that work in a narrow pH range, typically above pH 7.4^28,43^. The greater pH tolerance of hdmb was indispensable here because it avoided having to readjust the pH on a very small reaction volume after addition of the fragments as their TFA salts. The phenolic group of hdmb acts as an intramolecular catalyst of acyl transfer^33^. We considered that the higher pKa of the lysine ε−amine compared to the terminal α-amine group explained the failure of Tmb to assist ligation at the lysine side-chain. The pKa difference may presumably explain why most previous attempts at auxiliary-assisted ubiquitylations were performed between Gly_75_-Gly_76_ of Ub rather than directly on the isopeptide bond even though synthesis of the lysine building block with an auxiliary attached to the side-chain is not much more challenging than for glycine.

The hdmb auxiliary was designed to be stable to the deprotection conditions of peptide cleavage from the resin but removable after ligation (the benzylamine/benzylamide safety-catch)^25^. In our hands removal of related backbone amide substitution such as the 2-hydroxy-4-methoxybenzyl (Hmb) becomes more difficult with increasing chain-length especially from unprotected peptides. Therefore we treated the 18.7 KDa NEMO_CoZi_hdmb-Ub with TFA/TMSBr to remove the hdmb auxiliary. With this method we were certain of complete removal of the auxiliary as TFA fully solvates proteins. As has been demonstrated for many other large synthetic multidomain proteins, in our hands refolding was unproblematic as monitored by CD (Fig. 6a). This acid treatment is compatible with most other PTMs besides ubiquitylation such as phosphorylation^44^. Although there has been a focus towards developing what are considered milder deprotection conditions we observed no problems with this acid deprotection and the improvement in installation and ligation of the auxiliary more than compensated for any theoretical disadvantage.

The fully chemically characterised NEMO_CoZi_-Ub acted as a substrate for HOIP. This shows that the RBR domain of HOIP is sufficient to catalyse the formation of a linkage between Ub primed NEMO and a second molecule of Ub (Fig. 7b). Thus, in contrast to ab initio ubiquitylation, RBR linear chain elongation of NEMO is independent of HOIL-1. The result demonstrates that the synthesis of linear chains, which are anchored to its target protein does not require additional structural elements outside the catalytic core of HOIP. Based on our findings we suggest that linear ubiquitylation of NEMO occurs in two stages: a priming step where Ub is attached to NEMO, which involves an enzymatic function of LUBAC outside the RBR-LDD domain of HOIP followed by a second phase of chain elongation, where Ub is linked to M1 of the primed Ub, which only requires the HOIP catalytic activity.

The second phase is independent of HOIL-1 and only requires HOIP. This activity might be important for balanced NF-κB signalling by maintaining the extent of linear Ub chains conjugated to NEMO thereby counteracting the deubiquitylation activity of DUBs such as Otulin or CYLD which terminate NF-κB activation^45^. Future cell-based assays will be required to elucidate to which extent HOIL-1 independent LUBAC activity contributes to a sustained activation of the NF-κB pathway. In addition, our biophysical experiments showed that NEMO_CoZi_ and NEMO_CoZi_-Ub have similar affinities for di-Ub. This implies that NEMO modification with ubiquitin and binding of linear ubiquitin are not mutually exclusive. We therefore suggest that LUBAC mediated activation of IKK via NEMO ubiquitylation does not require dissociation from the Ub binding platform of the activated receptor-signalling complex. It is interesting to note that the melting point of NEMO_CoZi_ is shifted to higher temperature in its ubiquitylated form, thereby stabilising the coiled-coil (Supplementary Fig. 9). The increased structural rigidity of the CoZi domain might contribute to the conformational rearrangement, which takes place upon activation by M1-linked polyubiquitin^46^.

In summary, we have presented a short synthesis of the hdmb auxiliary and its easy addition to the lysine side-chain, enabling rapid access to site-specifically ubiquitylated NEMO. We have shown that priming and elongation are separate events located on different catalytic sites, reflecting the modular nature of the LUBAC complex. We are using this direct and specific ubiquitylation tactic to further explore the NFκB pathway. Hdmb is especially suited to three-piece ligations, required for larger targets, as the conditions of ligation are complementary and orthogonal to classical NCL^28,47^ and therefore potentially with mercaptolysine assisted ligation. The promising ligation properties of the hdmb auxiliary will be further explored with more sterically challenging ligations.

## Methods

### 2-hydroxy-3,4-dimethoxy-6-(4-methoxybenzylthio) benzaldehyde 3

Initially, commercially available 2,3,4-trimethoxy-6-nitrobenzaldehyde **1** (CAS: 52978–83–3) (Fluorochem) was used. For all subsequent preparations 2,3,4-tri-methoxybenzaldehyde (20 g, 101.9 mmol) in glacial acetic acid (50 mL) was cooled to −15 °C, 70% conc. HNO_3_ (50 mL) was slowly added and the mixture stirred at 0 °C for 1 h. The reaction mixture was poured into ice-water (350 mL) and the yellow precipitate filtered off, washed with cold water, and dried in a vacuum desiccator. Recrystallisation yielded **1** (8.59 g, 35%) as pale yellow crystals, mp 83–84 °C (EtOAc-hexanes). ^1^H NMR (400 MHz, CDCl_3_): *δ* 10.21 (s, 1H), 7.27 (s, 1H), 3.95 (s, 3H), 3.94 (s, 6H).

To a cold solution of **1** (4.83 g, 20 mmol) and 4-methoxybenzylmercaptan (3.08 g, 20 mmol) in DMF (40 mL) was added dropwise a solution of KOH (2.02 g, 36 mmol) in water (5 mL). The mixture was heated (80 °C, 6 h) and the resulting dark red solution poured into ice-water (350 mL). The brown precipitate was filtered, washed with cold water and dried. Recrystallisation yielded 2,3,4-trimethoxy-6-(4-methoxybenzylthio)benzaldehyde **2** (3.32 g, 48%) as light brown crystals, mp 150–152 °C (EtOAc-hexanes). ^1^H NMR (400 MHz, CDCl_3_): *δ* 10.32 (s, 1H), 7.32 (q_AA´BB´_, *J* = 8.7 Hz, 2H), 6.83 (q_AA´BB´_, *J* = 8.7 Hz, 2H), 6.56 (s, 1H), 4.08 (s, 2H), 3.96 (s, 3H), 3.81 (s, 3H), 3.79 (s, 3H), 3.77 (s, 3H).

A solution of **2** (3.32 g, 9.5 mmol) in dry CH_2_Cl_2_ (190 mL) was cooled to 0 °C and boron tribromide dimethyl sulfide complex (5.94 g, 19 mmol) added. The reaction was monitored by TLC [EtOAc-hexanes (3:7)]. After 3 h the bright red solution was washed with water (100 mL), evaporated, diluted with EtOAc (180 mL), washed with saturated aq. NaHCO_3_, brine, dried (MgSO_4_) and evaporated. Chromatography [EtOAc-hexanes (3:7)] yielded **3** (2.41 g, 76%) as light brown crystals, mp 123–124 °C (EtOAc-hexanes). ^1^H NMR (400 MHz, CDCl_3_): *δ* 12.19 (s, 1H), 10.17 (s, 1H), 7.04 (q_AA′BB′_, *J* = 8.6 Hz, 2H), 6.78 (q_AA′BB′_, *J* = 8.6 Hz, 2H), 6.49 (s, 1H), 3.97 (s, 2H), 3.86 (s, 3H), 3.81 (s, 3H), 3.76 (s, 3H) (Supplementary Fig. 10). Elemental analysis calc. C_17_H_18_O_5_S: C, 61.06; H, 5.43. Found: C, 61.22; H, 5.39.

### Peptide synthesis

Peptides were prepared by standard automated solid-phase synthesis using DIC/HOBt activation for Fmoc/t-Bu chemistry (CS Bio 336 automated synthesizer) except for LAPAG-*MPAL* prepared by HF-free Boc chemistry^44^. Couplings of amino acids were carried out with a five-fold excess of activated amino acid for a minimum of 45 min. Unless otherwise indicated, cleavage from the resin was performed by treatment of the peptide-resin with a cleavage cocktail containing TFA and as scavengers, triethylsilane (TES), H_2_O, ethanedithiol (EDT) in the ratio 94.5:2.5:2.5:0.5 (v/v) for 1.5 h. The cleavage cocktail was filtered, the filtrate sparged (N_2_) and the peptide precipitated (Et_2_O, Na-dried, −20 °C) and freeze-dried. Peptides were purified by semi-preparative HPLC on a RP-C18 column (22 × 250 mm, Vydac) using linear gradients of CH_3_CN in 0.1% TFA/H_2_O with a flow rate of 15 mL.min^−1^. Peptide analysis by HPLC was performed with a RP-C18 column (Phenomenex) or Diphenyl column (Vydac) using linear gradients of CH_3_CN in 0.1% TFA/H_2_O with a flow rate of 1 mL.min^−1^. HPLC gradients are given with A = 0.1% TFA in H_2_O and B = 0.1% TFA, 10% H_2_O, 90% CH_3_CN. Detection was performed at 214 nm. Peptides were characterised by MALDI-TOF MS on a BRUKER microflex using CHCA matrix (10 mg.mL^−1^ in CH_3_CN/H_2_O/TFA, 50:50:0.1) or by LCMS on a Shimadzu 2010EV.

### Synthesis of NEMO_298–308_hdmb (*H*-ADIYK(hdmb)ADFQAE-*NH_2_*)

The peptide was synthesised on Rink-amide resin (250 mg, 0.1 mmol) with standard Fmoc amino acids except for Lys^302^ (Fmoc-Lys(Alloc)-OH) and Ala^298^ (Boc-Ala-OH). A sample of peptide-resin (5 mg) was treated with TFA and scavengers and the peptide isolated as described above to check the peptide (Fig. 3). Lys^302^ was deprotected by treatment of the peptide-resin with Pd(PPH_3_)_4_ (29 mg, 0.025 mmol), PhSiH_3_ (308 μL, 2.5 mmol) in dry CH_2_Cl_2_ under Argon. Peptide-resin was washed with CH_2_Cl_2_ and DMF, followed by DMF containing diethyldithiocarbamic acid sodium salt trihydrate (0.5% w/v), and DIEA (0.5% v/v) to complex out residual palladium and washed again with DMF. Auxiliary **3** (33.4 mg, 0.1 mmol) was added with minimum DMF to the peptide-resin (0.1 mmol). After agitation (15 min) and washing (DMF), NaBH_4_ (10 mg, 0.26 mmol) was added and left for 5 min. Peptide-resin was finally washed (DMF) and dried. NEMO_298–308_hdmb-Mob was cleaved from the resin as described above. The crude peptide (typically 10 mg scale) was treated for 2 h at rt with a mixture of TFA/TMSBr/thioanisole/EDT (1:0.1:0.05:0.025; 8 mL) to remove the Mob protecting group. TFA was removed by sparging (N_2_) and the peptide precipitated (Et_2_O, Na-dried, −20 °C). Semipreparative HPLC of the pooled samples (RP-C18, 5–50% B in 30 min, 15 mL/min) yielded NEMO_298–308_hdmb (26 mg, 16.4 μmoles, 16.4%, purity >95%). The peptide was characterised by MALDI-TOF MS in positive ion linear mode: *m/z* = 1467.9 [M + H]^+^, calc. = 1467.6 (Fig. 3).

### Ligation of NEMO_298–308_hdmb and LAPAG-MPAL

NEMO_298–308_hdmb (1 mg, 0.59 μmol, final concentration 5 mM) was dissolved in 115 μL of degassed 200 mM sodium phosphate buffer, 2 mM EDTA, 6 M guanidine.HCl, 10 mg/mL MPAA, 15 mg/mL TCEP, pH 7.5. This solution was added to LAPAG-MPAL (0.64 mg, 0.86 μmole, final concentration 7.5 mM) and the mixture was kept at 40 °C. The final pH of the reaction mixture was 7. The ligation was monitored by analytical HPLC and was complete in 6 h 30 min (Supplementary Fig. 1). The reaction mixture was freeze-dried. The ligation product NEMO_298–308_hdmb-LAPAG, isolated from analytical HPLC, was characterized by MALDI-TOF MS in the positive ion reflector mode: *m/z* = 1876.3 [M + H]^+^, calc = 1876.9. The lyophilized mixture was treated at rt for 1 h 30 min with 1 mL of TFA/TMSBr/thioanisole/EDT (1:0.05:0.05:0.025) to remove hdmb. The TFA was removed by sparging under a stream of N_2_ and the peptides precipitated in cold Et_2_O. Purification by HPLC (RP-C18, 0 to 20% B, 1 mL/min) yielded NEMO_298–308_-LAPAG (0.62 mg, 0.325 μmoles, 55% overall yield for combined ligation reaction and auxiliary removal, purity > 95%). The peptide was characterized by MALDI-TOF MS in the positive ion reflector mode: *m/z* = 1679.3 [M + H]^+^, calc = 1679.8.

### Synthesis of Ub-αMeCys

(*H*-MQIFVKT**LT**GKT**IT**LEVEPSDTIENVKAKIQDKE GIPPDQQRLIFA**G**KQLED**G**RT**LS**DYNIQKE**ST**LHLVLRLRGGαMeC-*OH*): The peptide was synthesized as described above on Wang resin (0.1 mmol, 500 mg) loaded with Fmoc-αMeCys(Trt)-OH^35^, with the addition of pseudoproline or Hmbglycine at the positions indicated in bold in the sequence above (after El Oualid et al.^36^) yielding 3.2 g of dried peptide-resin. Cleavage was performed as previously described (typically, 5 mL TFA cleavage mixture was used to cleave 100 mg peptide-resin, 3.125 μmol). Crude peptide was dissolved in 20% buffer B in A and freeze-dried. Purification by HPLC (RP-C18, 25–45% B in 30 min, 15 mL/min) yielded Ub-αMeCys (9.1 mg obtained from 100 mg peptide-resin, 0.895 μmole, 28.6% yield, purity >95%). The peptide was characterized by MALDI-TOF MS in the positive ion linear mode: *m/z* = 8682 [M + H]^+^, calc = 8682 (Fig. 4).

### Synthesis of Ub-MES thioester by *N,S* acyl transfer and thioester exchange from Ub-αMeCys

Pure Ub-**α**MeCys (3.8 mg, 0.374 μmol, final concentration 0.1 mM) was dissolved in degassed 200 mM sodium phosphate buffer, 2 mM EDTA, 6 M guanidine.HCl, 5 mg/mL TCEP (17.5 mM), 100 mg/mL MesNa (610 mM), pH 4.5 to 5. The reaction mixture was kept at 50 °C. Ub-MES and starting material Ub-**α**MeCys co-eluted on all HPLC columns and gradients attempted. Monitoring the reaction was possible by treatment of small samples of the reaction mixture with hydrazine, the hydrazide product formed from Ub-MES thioester was separable on C18 and indicated the progress of the reaction. The reaction was also monitored by LC-MS and was complete after 7 h. Purification by HPLC (RP-C18, 25–45% B in 30 min, 15 mL/min) yielded Ub-MES thioester (3.5 mg, 0.345 μmole, 90% yield). The peptide was characterized by MALDI-TOF MS in the positive ion linear mode: *m/z* = 8689 [M + H]^+^, calc = 8689 (Supplementary Fig. 2).

### Synthesis of Ub-MPAA thioester by *N,S* acyl transfer and thioester exchange from Ub-αMeCys

Pure Ub-**α**MeCys (7.1 mg, 0.699 μmol, final concentration 0.2 mM) was dissolved in 3.5 mL degassed 200 mM sodium phosphate buffer, 2 mM EDTA, 6 M guanidine.HCl, 10 mg/mL TCEP (17.5 mM), 25 mg/ml MPAA (150 mM), pH 4.5–5. The reaction mixture was kept for 8 h at 50 °C. In this case the exchange reaction could be easily monitored by analytical HPLC (Supplementary Fig. 3). TFA (50 μL) was added to acidify and quench the reaction mixture (final pH 2) and the mixture was extracted with Et_2_O to remove the MPAA thiol additive. Purification by HPLC of the aqueous phase (RP-C18, 30–45% B in 30 min, 5 mL/min) yielded Ub-MPAA thioester (2.4 mg, 0. 235 μmole, 34% yield). The peptide was characterized by MALDI-TOF MS in the positive ion linear mode: *m/z* = 8715 [M + H]^+^, calc = 8715 (Supplementary Fig. 4).

### Ligation of NEMO_298–308_hdmb and Ub-MES

NEMO_298–308_hdmb (0.34 mg, 0.2 μmol, final concentration 4 mM,) was dissolved in 50 μL of degassed 200 mM sodium phosphate buffer, 2 mM EDTA, 6 M guanidine.HCl, 15 mg/mL TCEP, pH 7.5. This solution was added to Ub-MES thioester (1 mg, 0.1 μmol, final concentration 2 mM) and the mixture kept at 40 °C. The final pH of the reaction mixture was 6.5. The ligation was monitored by HPLC and was complete around 8 h (Fig. 4). The mixture was freeze-dried. The ligation product NEMO_298–308_hdmb-Ub, isolated from analytical HPLC, was characterised by MALDI-TOF MS in the positive ion linear mode: *m/z* = 10012 [M + H]^+^, calc = 10015.5. The lyophilised mixture was treated for 1 h 30 min at rt with 1 mL of TFA/TMSBr/thioanisole/EDT (1:0.05:0.05:0.025). The TFA was removed by sparging under a stream of N_2_ and the peptides precipitated in cold Et_2_O. Purification by HPLC (Diphenyl, 10–50% B in 30 min, 1 mL/min) yielded NEMO_298–308_-Ub (0.65 mg, 0.057 μmoles, 58% overall yield for combined ligation and auxiliary removal, purity >98%). The peptide was characterised by MALDI-TOF MS in the positive ion linear mode: *m/z* = 9818 [M + H]^+^, calc. Av. = 9817 (Fig. 4).

### Ligation of NEMO_298–308_hdmb and Ub-MPAA

NEMO_298–308_hdmb (0.6 mg, 0.35 μmol, final concentration 2 mM) was dissolved in 177 μL of degassed 200 mM sodium phosphate, 2 mM EDTA, 6 M guanidine.HCl, 15 mg/mL TCEP, 0.5 mg/mL MPAA, pH 7.5. 40 μL of this solution was added to Ub-MPAA thioester (0.41 mg, 0.04 μmol, final concentration 1 mM) and the mixture was kept at 40 °C. The final pH of the reaction mixture was 6.9. The ligation was complete in 2 h. Removal of the auxiliary was performed as previously described.

### Synthesis of NEMO_CoZi_hdmb

(Biotin-EDLRQQLQQAEEALVAKQELIDKLKEEAEQHKIVMETVPVLKAQADIYK(hdmb)ADFQAERHAREKLVEKKEYLQEQLEQLQREFNKL-*NH_2_*): The peptide was synthesised on Rink-amide ChemMatrix resin (Aldrich) (100 mg, 0.05 mmol) with standard Fmoc amino acids except for Lys^302^ (Fmoc-Lys(Alloc)-OH). The weight of the dried peptide-resin at completion of the synthesis was 730 mg. Alloc removal was performed as described above using Pd(PPh_3_)_4_ (15 mg, 0.013 mmol), PhSiH_3_ (150 μL, 1.22 mmol) in dry CH_2_Cl_2_ (10 mL). A sample of the peptide-resin was cleaved and analysed by HPLC and MALDI-TOF MS (Supplementary Fig. 5). To the peptide-resin (0.025 mmol) in minimal DMF (swelling volume) was added auxiliary **3** (15 mg, 0.045 mmol) followed by treatment with NaBH_4_ (38 mg, 1 mmol) as described above (Supplementary Fig. 6). The peptide was cleaved from the resin, purified by HPLC (RP-C18, 20–60% B in 30 min, 15 mL/min) yielding NEMO_CoZi_hdmb-Mob (9.4 mg, 0.772 μmole, 3%) and characterized by LC-MS (*m/z* = 10462 [M + H]^+^, calc = 10462). NEMO_CoZi_hdmb-Mob was treated for 2 h at rt with 3 mL of a mixture of TFA/TMSBr/thioanisole/EDT (1:0.1:0.05:0.025). The TFA was removed by sparging (N_2_) and the peptide precipitated (Et_2_O, Na-dried, −20 °C). Purification by HPLC (RP-C18, 20–60% B in 30 min, 15 mL/min) yielded NEMO_CoZi_hdmb (4.8 mg, 0.398 μmole, 51.5% yield, purity >95%). The peptide was characterised by LCMS: *m/z* = 10342 [M + H]^+^, calc. Av. = 10342 (Supplementary Fig. 7).

### Ligation of NEMO_CoZi_hdmb and Ub-MPAA thioester

NEMO_CoZi_hdmb (1.32 mg, 0.1085 μmol, final concentration 1 mM) was dissolved in 100 μL of degassed 200 mM sodium phosphate, 2 mM EDTA, 6 M guanidine.HCl, 15 mg/mL TCEP, 0.5 mg/mL MPAA, pH 7.5. This solution was added to Ub-MPAA thioester (1.39 mg, 0.134 μmol) and the mixture was kept at 40 °C. After 2 h, further Ub-MPAA thioester (0.5 mg) was added. The final pH of the ligation mixture was 6.5. The ligation was monitored by gel electrophoresis (Fig. 5a). After 6 h, the reaction mixture was diluted with 100 μL H_2_O and freeze-dried. It was treated for 2 h with 150 μL of TFA/TMSBr/thioanisole/EDT (1:0.05:0.05:0.025). TFA was removed by sparging under a stream of N_2_ and the protein precipitated in cold Et_2_O. The crude mixture was dissolved in 150 μL of degassed 200 mM sodium phosphate, 2 mM EDTA, 6 M guanidine.HCl, 5 mg/mL TCEP and purified by HPLC (RP-C18, 20–55% B in 30 min, 1 mL/min). To calculate the yield, the ligation was repeated in the same conditions using NEMO_CoZi_hdmb (2.15 mg, 0.177 μmol) and Ub-*MPAA* thioester (3.5 mg, 0.34 μmol). Auxiliary removal and HPLC purification yielded NEMO_CoZi_-Ub (1.46 mg, 0.0667 μmoles, 38% overall yield for combined ligation and auxiliary removal). NEMO_CoZi_-Ub was characterised by MALDI-TOF MS in the positive ion linear mode: *m/z* = 18688 [M + H]^+^, calc. Av. = 18690. (Fig. 5b).

### LC-MSMS analysis of NEMO_CoZi_-Ub

NEMO_CoZi_-Ub was resuspended in 50 mM NH_4_HCO_3_ and digested o/n using trypsin. Digested peptides were dried using a centrifugal SpeedVac and resuspended in MS solvent (3% CH_3_CN, 0.1% TFA). Tryptic peptides were analysed on a Q Exactive coupled to an RSLCnano3000 (Thermo Scientific). Peptides were resolved using a 50 cm EASYspray column with a gradient rising from 3 to 40% CH_3_CN over 23 min. MS spectra were acquired from *m/z* 300–1500 with peptides selected for fragmentation in a DDA fashion. MSMS spectra were acquired at 17,500 fwhm. Raw files were processed in PEAKS studio using the PEAKS search engine. Data was searched against a database consisting of the synthetic NEMO sequence in an *E.coli* K12 database and a database of common contaminants. A PEAKS PTM search was performed with confident modification sites requiring a minimum ion intensity of 5% (Supplementary Fig 8).

### Circular dichroism measurements

CD spectra and melting curves were recorded on a Jasco J-815 spectropolarimeter fitted with a cell holder thermostatted by a CDF-426S Peltier unit. CD measurements were made in PBS buffer pH 7.4 using fused silica cuvettes with 1-mm or 10-mm path lengths (Hellma, Germany).

Far-UV CD spectra were recorded with 2 nm bandwidth and baseline corrected by subtraction of the appropriate buffer spectrum. CD intensities are presented as the CD absorption coefficient calculated on a molar basis (Δε_M_)^48^. Secondary structure content was estimated by methods described by Sreerama and Woody^49^. Thermal unfolding curves were obtained by monitoring the ellipticity at 222 nm using 1- or 10-mm path length cuvettes and a heating rate of 2 °C/min over the temperature range 2–80 °C. The transition mid-point temperature was estimated from the point of inflection, by determining the maximum of the first derivative of the melting curves.

### Di-Ub binding measurements

Di-Ub binding to immobilized NEMO_CoZi_ and NEMO_CoZi_-Ub were measured on an Octet RED biolayer interferometer (Pall ForteBio Corp., Menlo Park, CA, USA). The Biotinylated NEMO_CoZi_ was immobilized on streptavidin biosensors (Pall ForteBio Corp., Menlo Park, CA, USA) at a concentration of ~2 μg/mL. The binding of di-Ub (at 2–225 μM) to immobilized NEMO was measured at 25 °C with a 100 second association step followed by a 100 s dissociation step. The buffer was 10 mM HEPES (pH 7.4), 150 mM NaCl, 3 mM EDTA and 0.005% Tween-20. The equilibrium dissociation constant was determined by analysis of the variation of the instrument response as a function of di-Ub concentration (Fig. 7a).

### Protein expression and purification

Human Ube1 and ubiquitin were cloned into pET-24 and expressed in BL21(DE3). Proteins were isolated by Ni affinity chromatography and subsequently purified by anion exchange chromatography followed by size exclusion chromatography (Superdex S75/S200). Human UBE2D1was cloned into pET-49 and HOIP-RBR (residues 697–1072) was cloned into pGex-6P1. After expression in BL21(DE3) proteins were isolated by GST affinity chromatography. The GST fusion was cleaved with PreScission Protease and subsequently removed by size exclusion chromatography (Superdex S75/S200). All purification steps were monitored by SDS gel electrophoresis. The protein concentrations were determined by UV-VIS spectroscopy at 280 nm using theoretical extinction coefficients calculated by ProtParam (http://web.expasy.org/protparam/) Purified samples were concentrated and stored in 50 mM HEPES pH 7.4, 150 mM NaCl and 1 mM DTT.

### In vitro ubiquitylation assay

1 μM Ube1, 5 μM UBE2D1, 5 μM of HOIP-RBR, 20 μM His-Ub, and 10 μM synthetic NEMO_CoZi_-Ub were mixed in 50 mM HEPES pH 7.5, 150 mM NaCl and 20 mM MgCl_2_. NEMO_CoZi_-Ub ubiquitylation with His-Ub was started by adding 10 mM ATP. The reaction was incubated at 25 °C for 1 h and samples were taken at set time intervals (0 min (without ATP), 5 min, 15 min, 30 min, 60 min), quenched with DTT and analysed by SDS-PAGE (Fig. 7b).

## Supplementary Material

**Supplementary information** accompanies this paper at https://doi.org/10.1038/s42004-019-0211-7.

sup_info

## Figures and Tables

**Fig. 1:**
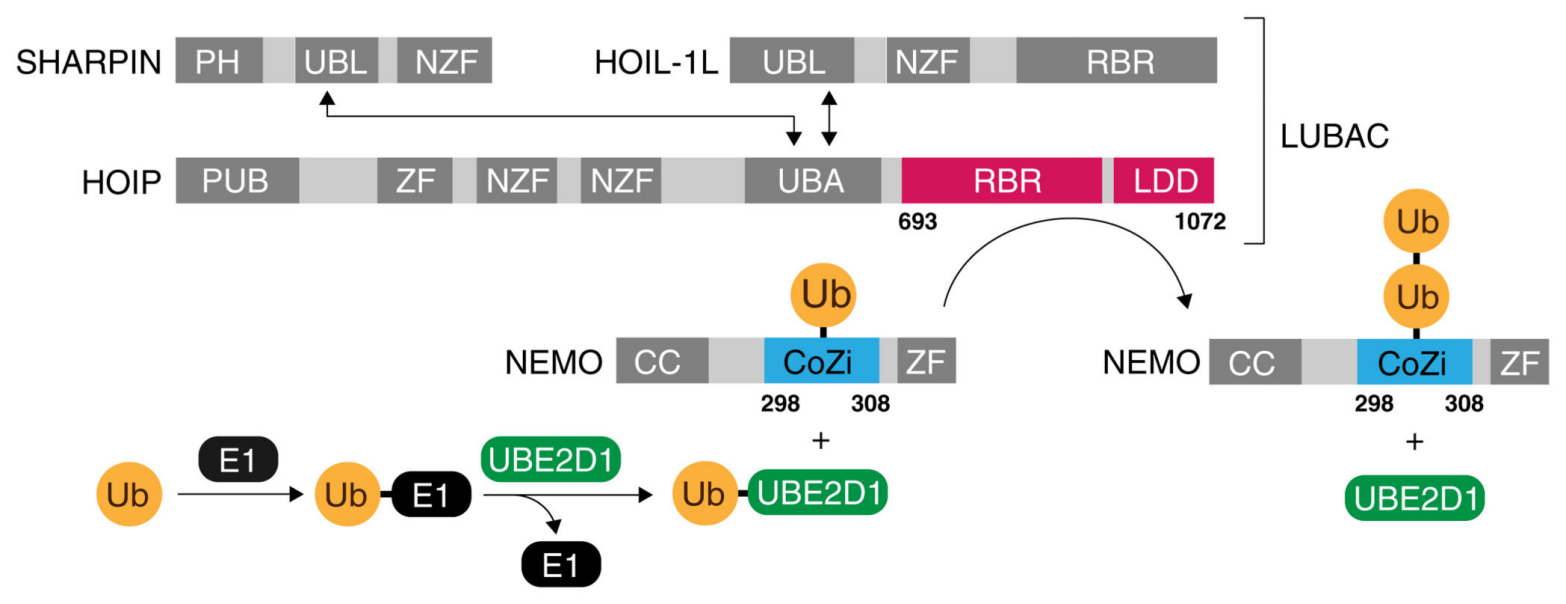
Schematic overview of the ubiquitylation pathway of NEMO. Ubiquitin is activated by an E1 enzyme (black) and passed onto the E2 enzyme UBE2D1 (green) in a transthiolation reaction. LUBAC, which consists of the three proteins HOIP, HOIL-1L and SHARPIN catalyses the formation of a peptide bond between the N-terminus of a ubiquitin molecule which has been conjugated to NEMO and the C-terminus of the activated ubiquitin provided by UBE2D1. Interaction between the UBL (ubiquitin like) domains of HOIL-1L and SHARPIN with the UBA (ubiquitin associated) domain of HOIP is required for full activity under in vivo condition. The formation of di-ubiquitin conjugated to lysine 303 was reconstituted under in vitro conditions using the minimal catalytic core of LUBAC, consisting of the RBR (RING in between RING) and LDD (linear chain determining) domain of HOIP (residues 693–1072, shown red) and the fully synthetic CoZi domain of NEMO (residues 298–308, shown in blue) ligated to ubiquitin (orange). The domains of HOIP and the two proteins HOIL-1L and SHARPIN, which are not present in the reconstitution system are shaded in grey (PH peckstrin homology, NZF Npl4 zinc finger, PUB PNGase/UBA/UBX, ZF zinc finger)
