# Supplementary material for: Auxiliary-assisted chemical ubiquitylation of NEMO and linear extension by HOIP
Source: Commun Chem. Author manuscript; Available in PMC 2020 Jan 15. (PMC6962051; doi:10.1038/s42004-019-0211-7)
Supplement: sup_info [file EMS85226-supplement-sup_info.pdf]

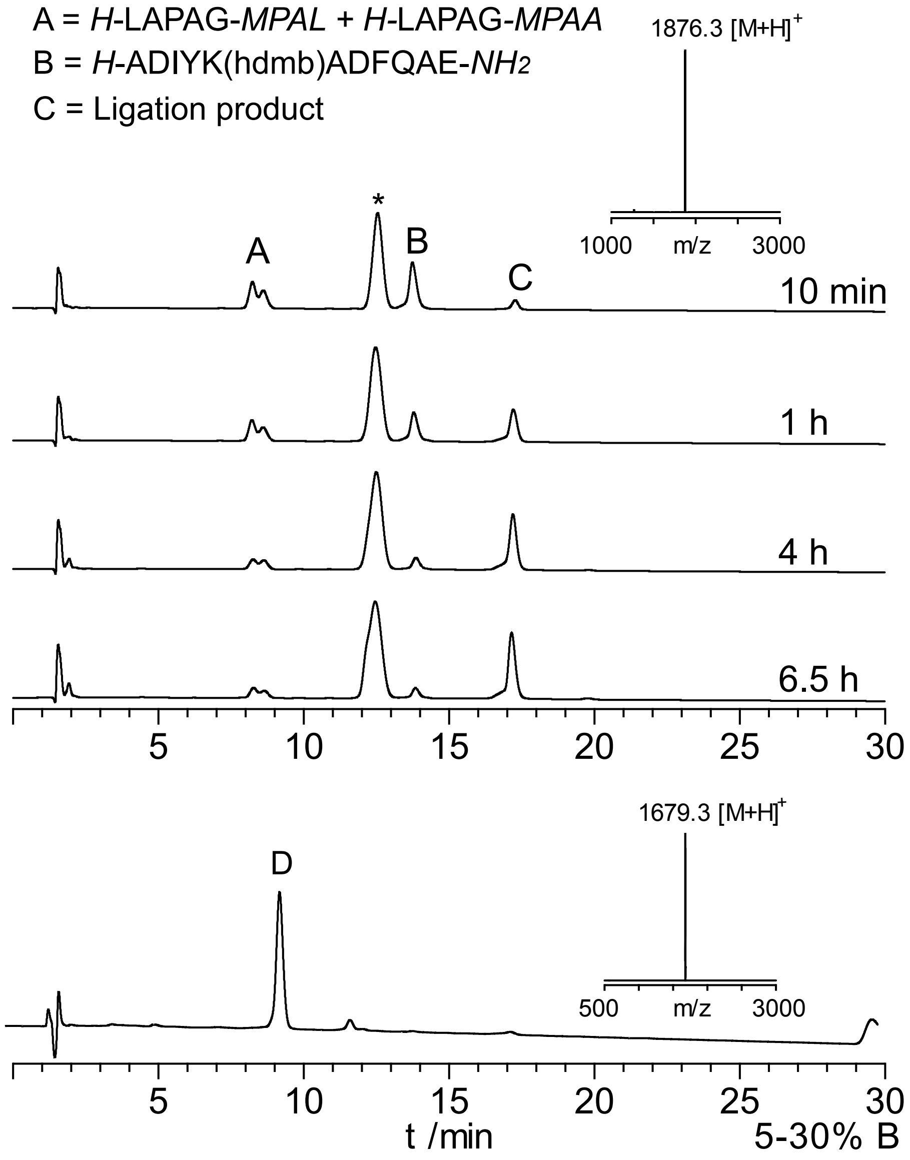

Supplementary Figure 1. Model ligation of $NEMO_{298\text{-}308}$hdmb with LAPAG-MPAL. Time-course monitoring of ligation reaction by analytical HPLC, RP-C18, (2 μL aliquots of the reaction mixture diluted in 10 μL H_2O were injected) and MALDI-TOF MS of purified peptide C (ligation product with hdmb auxiliary), m/z = 1873.6 $[M+H]^+$, calc. = 1876.9, * = MPAA. Analytical HPLC of crude product after hdmb removal from purified peptide C and MALDI-TOF MS, m/z = 1679.3 $[M+H]^+$, calc. = 1679.8, D = ligation product without hdmb.

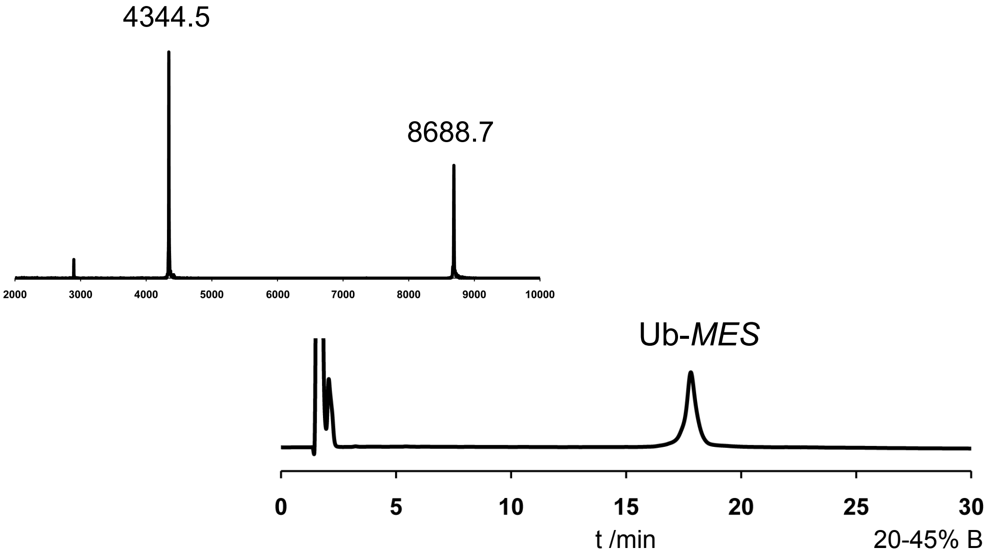

Supplementary Figure 2. Synthesis of Ub-MES thioester by N,S acyl transfer and thioester exchange from Ub-**α**MeCys. Analytical HPLC (RP-C18) and MALDI-TOF MS of Ub-MES, m/z = 8689 [M+H]+,calc. Av. = 8689.

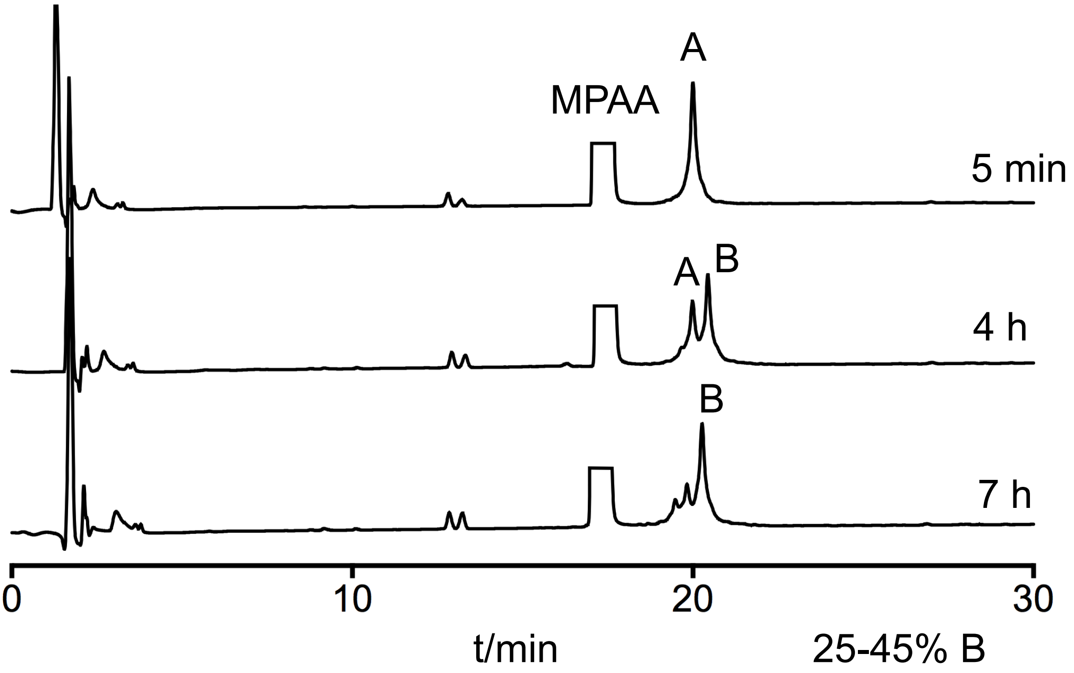

Supplementary Figure 3. Time course of Ub-MPAA thioester synthesis by *N,S* acyl transfer and thioester exchange from Ub-αMeCys monitored by analytical HPLC (RP-C18) injecting 10 μL aliquots of the reaction mixture. A = Ub-αMeCys, B = Ub-MPAA thioester.

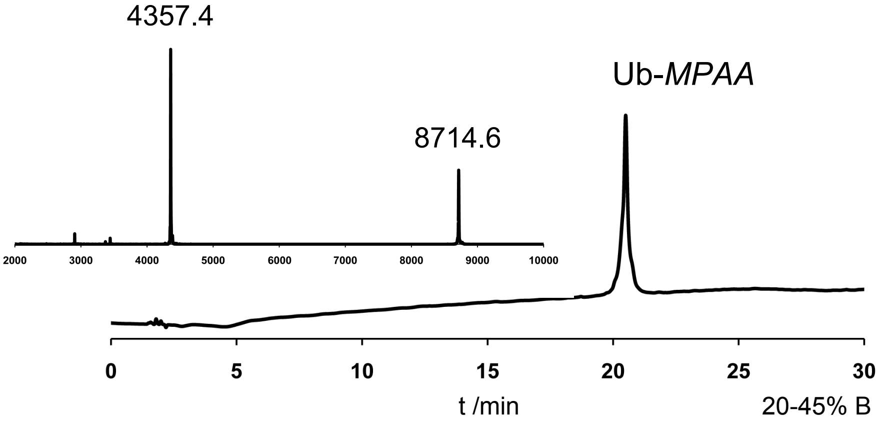

Supplementary Figure 4. Analytical HPLC (RP-C18) and MALDI-TOF MS of purified Ub-MPAA, m/z = 8715 $[M+H]^+$, calc. Av. = 8715.

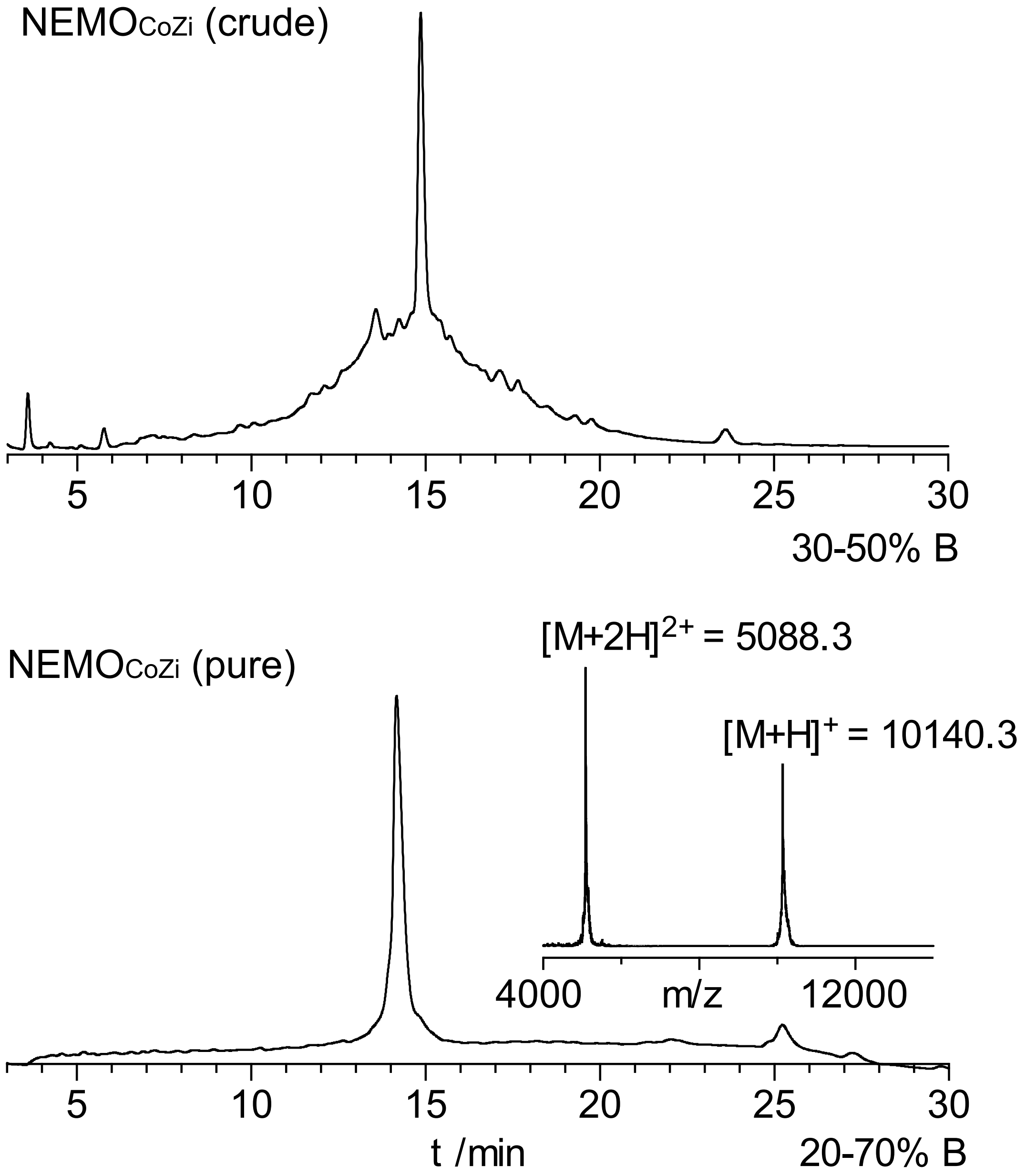

Supplementary Figure 5. Analytical HPLC (RP-C18) of crude and purified $NEMO_{CoZi}$ (after removal of Alloc). *Biotin*-EDLRQQLQQAEEALVAKQELIDKLKEEAEQHKIVMETVPV LKAQADIYKADFQAERHAREKLVEKKEYLQEQLEQLQREFNKL-*NH*$_2$.
Insert shows MALDI-TOF MS, m/z = 10140 $[M+H]^+$, calc. Av. = 10144.

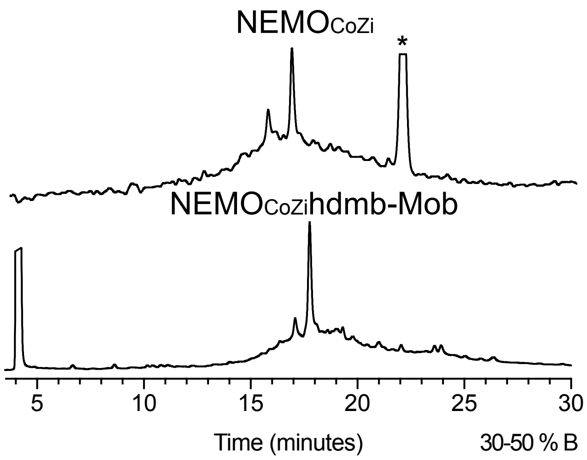

SupplementaryFigure 6. On-resin installation of the ligation auxiliary hdmb (**3**) into $NEMO_{CoZi}$. Analytical HPLC (RP-C18) of crude $NEMO_{CoZi}$ and $NEMO_{CoZi}$hdmb-Mob. * = scavenger

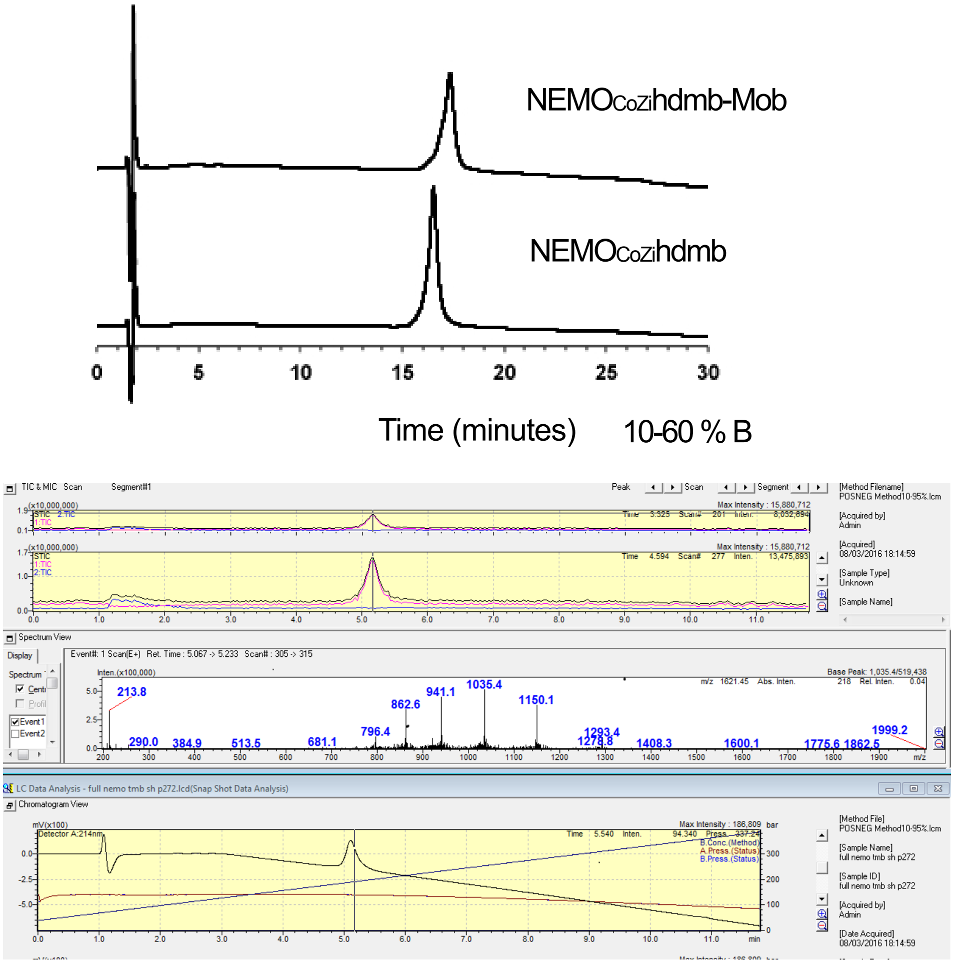

·

Supplementary Figure 7. Removal of Mob protecting group in solution. Analytical HPLC (RP-C18) of pure $NEMO_{CoZi}$hdmb-Mob and $NEMO_{CoZi}$hdmb and LCMS of $NEMO_{CoZi}$hdm m/z = 10342 $[M+H]^+$, calc. Av. = 10342.

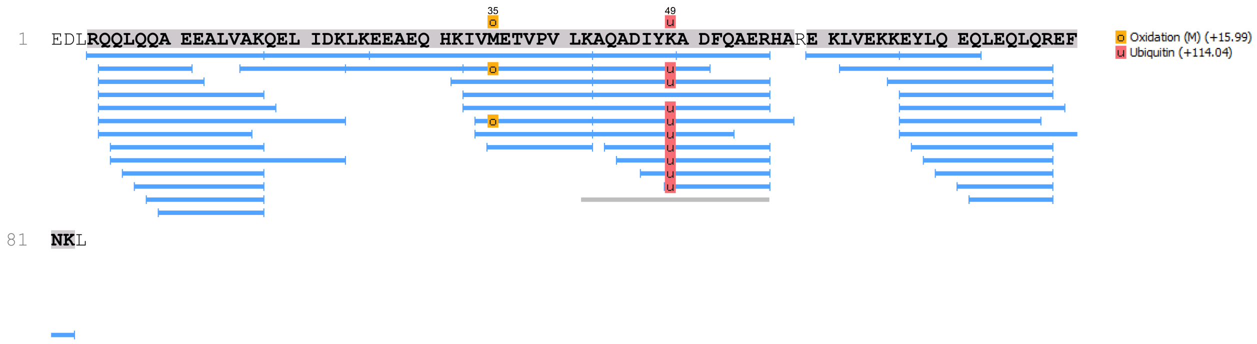

Supplementary Figure 8. Sequence coverage of NEMO$_{CoZi}$-Ub derived from LC-MSMS indicating ubiquitylation site (red). Oxidation of Met^{35} was also detected (orange).

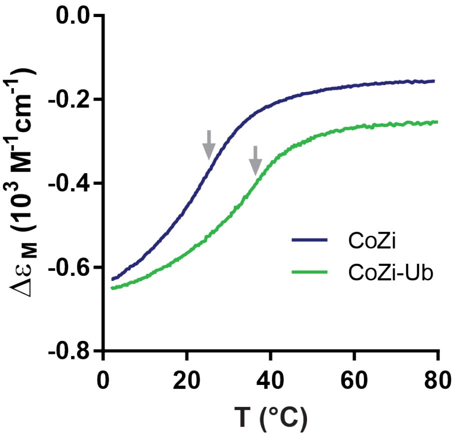

Supplementary Figure 9. Comparison of thermal melting curves of synthetic NEMO$_{CoZi}$-Ub (8 μM) and NEMO$_{CoZi}$ (16 μM), measured by circular dichroism at 222 nm. The melting temperatures (grey arrows) of NEMO$_{CoZi}$-Ub and NEMO$_{CoZi}$ are 36.4 °C and 24.9 °C, respectively.

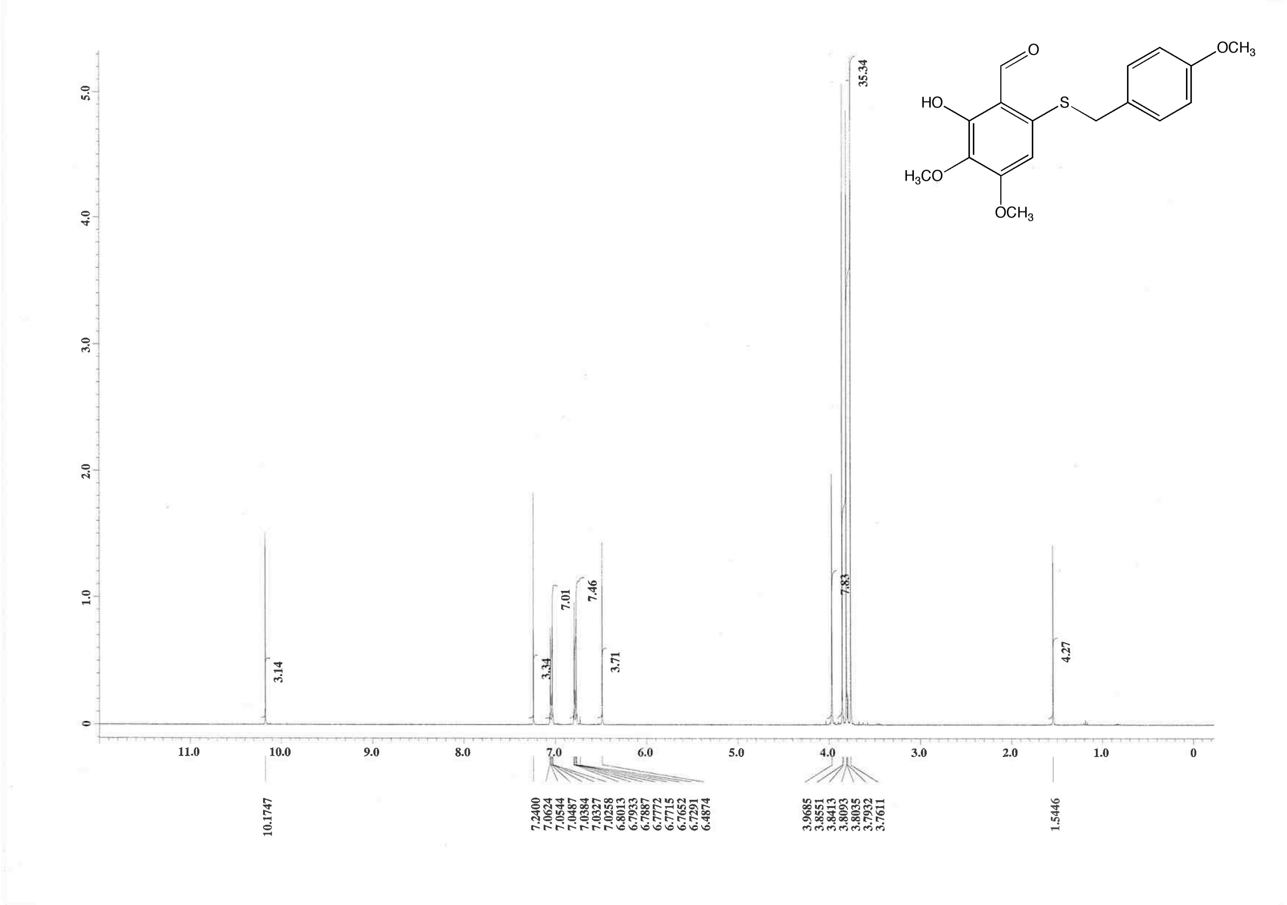

Supplementary Figure 10.^{1}H NMR of hdmb aldehyde **3**.
